# Both DNA global deformation and repair enzyme contacts mediate flipping of thymine dimer damage

**DOI:** 10.1038/srep41324

**Published:** 2017-01-27

**Authors:** Alexander Knips, Martin Zacharias

**Affiliations:** 1 Physik-Department T38, Technische Universität München, James-Franck-Str. 1, D-85748 Garching, Germany

## Abstract

The photo-induced cis-syn-cyclobutane pyrimidine (CPD) dimer is a frequent DNA lesion. In bacteria photolyases efficiently repair dimers employing a light-driven reaction after flipping out the CPD damage to the active site. How the repair enzyme identifies a damaged site and how the damage is flipped out without external energy is still unclear. Employing molecular dynamics free energy calculations, the CPD flipping process was systematically compared to flipping undamaged nucleotides in various DNA global states and bound to photolyase enzyme. The global DNA deformation alone (without protein) significantly reduces the flipping penalty and induces a partially looped out state of the damage but not undamaged nucleotides. Bound enzyme further lowers the penalty for CPD damage flipping with a lower free energy of the flipped nucleotides in the active site compared to intra-helical state (not for undamaged DNA). Both the reduced penalty and partial looping by global DNA deformation contribute to a significantly shorter mean first passage time for CPD flipping compared to regular nucleotides which increases the repair likelihood upon short time encounter between repair enzyme and DNA.

Irradiation of the cell with ultra-violet light can result in covalent dimer formation of neighboring pyrimidines in DNA^1^,^2^. The most common pyrimidine dimer is the cis-syn-cyclobutane pyrimidine dimer (CPD) lesion between adjacent thymines. If not repaired the CPD lesion is highly cytotoxic, mutagenic, and carcinogenic^1^,^2^. Bacteria and eukaryotic cells have evolved different strategies to recognize and repair CPD lessons and the related 6–4 photo product (6–4PP)^3^. In bacteria both CPD and 6–4PP lesions can be reversed by exposure to blue light employing a light-induced reaction catalyzed by a DNA photolyase. Repair enzyme binding of damaged DNA is specific. *E. coli* DNA photolyase binds a CPD thymine dimer containing DNA with a dissociation constant of 30 nM which is 75000 stronger than the binding to undamaged DNA^4^. However, the molecular mechanism of specific recognition and repair of CPD damaged DNA is still incompletely understood.

The crystal structure analysis of a CPD lesion in complex with *E. coli* DNA photolyase indicates an extra-helical conformation of the damage in order to fit into the enzyme active site and to allow a close contact with the enzyme bound excited FADH required for splitting the dimer^5^ (PDB structure: pdb:1TEZ). Although crystallized in the presence of an intact CPD lesion this structure has undergone photochemical repair of the damage (photochemical splittiing to form a product complex), it is likely to present a good model of the CPD substrate complex because structure determination at very low temperature (<100 K) and during short X-ray exposure is unlikely to allow major conformational changes. Furthermore, a subsequent structure determination of a related CPD repair enzyme succeeded in solving a complex with an intact CPD damage and this structure indicated a very similar placement of the CPD damage in the enzyme binding pocket^6^ as seen in the pdb:1TEZ structure. In contrast, the crystal structures and nuclear magnetic resonance (NMR) spectroscopy of isolated CPD damage containing DNA indicate an intra-helical state in the context of double stranded (ds)DNA^7^,^8^,^9^,^10^,^11^,^12^,^13^,^14^,^15^,^16^,^17^. In the intra-helical state the CPD lesion forms hydrogen bond contacts with bases on the opposite strand. However, the structural studies on CPD containing DNA indicate also a significant distortion of the DNA with respect to regular B-DNA due to the damage^7^. These structural alterations are also found in electron microscopy and gel electrophoresis studies of CPD damaged DNA^18^,^19^,^20^.

Different mechanisms of damage recognition by repair enzymes have been proposed^21^. In a passive conformational selection mechanism the repair enzyme relies on a random base flipping event which leads to subsequent binding and initiation of the repair process. In contrast, during an active (induced fit) process the repair enzyme binds first transiently forming an encounter complex (with the damaged site still in an intra-helical conformation) followed by the flipping process in the presence of the repair enzyme.

Recent Molecular Dynamics (MD) simulations comparing regular B-DNA and a CPD lesion in the same sequence context also indicated distortions caused by the CPD damage and increased DNA flexibility^22^,^23^,^24^ but during the simulations the damage remained intra-helical without any spontaneous flipping to a looped out state in 1 μs simulation time^22^. However, during simulations the CPD containing DNA adopted transiently conformations that come globally closer to the conformation observed in the crystal structure in complex with the photolyase repair enzyme (with extra-helical lesion) than regular undamaged DNA although the lesion remained in an intra-helical conformation^22^. The transiently sampled near bound conformations allow an initial preferential encounter recognition following mechanistically a conformational selection process. Such scenario also suggests a recognition mechanism largely based on the damage structure rather than detailed sequence of the DNA. The subsequent looping out transition of the CPD into the enzyme active site can then be considered as an induced fit step of the repair process. It is important to note that for other repair processes such step-wise recognition and repair mechanism is supported by crystal structures of encounter complexes. For example, in case of oxidatively damaged guanine to 8-oxo-guanine, encounter complexes with untwisted and strongly bend DNA and intra-helical damaged **8oxoG** have been structurally characterized^25^.

The presence of the enzyme can facilitate the flipping process due to specific protein-DNA contacts. However, it is also possible that binding induced deformation of the DNA into a structure that lowers the penalty for a flipping of the CPD lesion contributes. The induced fit process is supported by the fact that all currently available structures (including solution NMR structures) in the absence of a repair protein indicate an intra-helical conformation of the CPD damage. In order to characterize the transition from the intra-helical to the extra-helical conformation of a CPD lesion it is possible to calculate the free energy change along an appropriate reaction coordinate to describe the transition during MD simulations. Such simulations have been used to study base pair opening in regular DNA^26^,^27^, mismatch opening^28^,^29^ and the recognition of methylated or damaged DNA^29^,^30^,^31^,^32^,^33^. These free energy simulations on flipping regular and chemically modified nucleo bases revealed free energy penalties of flipping between 8 kcal/mol–15 kcal/mol. However, a possible coupling of the flipping process and global deformations in DNA has so far not been investigated.

For the flipping of the cis,syn-cyclobutane pyrimidine dimer (CPD) inside an isolated duplex DNA^34^,^35^,^36^ a free energy change of approximately 6 kcal/mol–7.5 kcal/mol has been obtained^35^. It translates to an equilibrium partition or lowering of the accessible extra-helical CPD lesion by a factor of 10^−5^ to 10^−6^ again disfavoring a passive recognition mechanism. Most previous simulation studies have studied flipping processes of undamaged or damaged bases in isolated regular DNA or on a complex with a repair enzyme. However, the influence of the repair enzyme onto the conformational transition of the lesion towards an extra-helical state can be twofold. Firstly, binding of the enzyme can result in a deformation of the DNA (e.g. changes in bending and twisting) that may lower the barrier or penalty for a looping out process. Hence, part of the binding free energy is stored as DNA deformation and, in principle, such mechanism does not require any direct contacts between the damaged base and the protein. Secondly, direct protein-DNA contacts during encounter may mediate or facilitate the looping out process. In order to elucidate different contributions to the recognition and base flipping process in case of a CPD damage we perform Molecular Dynamics free energy energy simulations of an induced flipping process for several conformational and association states of damaged and undamaged DNA. It includes unrestrained double stranded (ds)DNA in the absence of repair enzyme, DNA deformed to a structure that mimics the protein induced deformation (but again without repair enzyme) and employing DNA in complex with the photolyase repair enzyme. Our study indicates that globally deforming the DNA towards the enzyme-bound structure alone contributes significantly to a lowering of the free barrier for CPD damage flipping. The deformation also results in a placement of the damaged nucleotides that reduces the pathlength for flipping. Contacts between protein and DNA further facilitate the flipping process. Comparison to the undamaged DNA helps to identify damage-specific contributions. In addition, by estimating the local diffusivity along the reaction coordinate the simulations also allowed us to estimate the kinetics of the process indicating a much more rapid flipping (factor 30) for the CPD damage compared to regular DNA in the global deformed state and in the presence of the bound repair enzyme. The results likely have implications for the recognition mechanism of other types of DNA damages since for most significant DNA deformations in complex with repair enzymes have been observed.

## Material and Methods

All molecular dynamics (MD) simulations were performed using the Amber12 suite of programs^37^ in explicit water (TIP3P)^38^ with a truncated octahedral box and a minimum distance of 10 Å between solute and box boundary. Potassium ions and chloride ions were included to neutralize the system and to adjust to physiological salt concentration of approximately 100 mM employing the Joung/Cheatham ion parameters for TIP3P water^39^,^40^. The simulations were carried out with the pmemd module of the Amber12 package using the ff99bsc0 and chi.OL3 force fields for nucleic acids^41^,^42^ and the ff99SB for proteins. The parameters by Spector *et al*. were used to describe the CPD damage^43^.

The simulation systems were first minimized by a steepest energy descent method (1500 steps) and heated (each step 100 ps) to 300 K in three steps of 100 K, then followed by gradual removal of the positional restraints from 25 kcal/(mol Å^2^) to 0.5 kcal/(mol Å^2^) (in 5 steps) and a 1 ns unrestrained equilibration at 300 K. All bonds between heavy atoms were constraint using SHAKE^37^ allowing a time step of 2fs in all simulations. Duplex DNA with the regular sequence d(5′-TCGGCTTCGCGC/5′-GCGCGAAGCCGA) or the equivalent sequence containing a central CPD damage (termed: = CPD=) d(5′-TCGGC = CPD = CGCGC/5′-GCGCGAAGCCGA) were used in all simulations identical to the sequence in the crystal structure of a DNA repair complex with the Escherichia coli (*E. coli*) DNA-photolyase (pdb:1TEZ^5^ with two additional 3′-G:C base pairs. The addition creates a DNA with a central damage and avoids fraying of the termini during simulations.

B-DNA starting structures of the isolated undamaged and damaged DNA duplexes were generated with the NAB (nucleic acid builder) tool of the Amber12 tools suite^37^. The structures are termed **TT**_**BDNA**_ and **CPD**_**BDNA**_, respectively, and contain the central bases in an intra-helical conformation. A second set of simulations was started from DNA conformations close to the enzyme bound form but intra-helical central CPD damage (or central TT nucleotides). The structures were generated by a simulation of (1 ns) starting from the B-DNA conformations and including positional restraints referenced the DNA backbone structure in the complex with the photolyase (in pdb:1TEZ). Positional restraints were used for the backbone atoms (P, C5′, C4′, C3′, O5′ and O3′) excluding the extra-helical bases and directly flanking nucleotides. The root-mean-square deviation (RMSD of heavy atoms) of the generated DNA conformations with respect to the enzyme bound structure was below 0.5 Å (excluding the central damage or TT nucleotides). The corresponding structures are termed **TT/CPD**_**1TEZ**_, respectively. Finally, MD simulations were also initiated from the complete pdb:1TEZ structure where DNA is in complex with the DNA-photolyase repair enzyme and the central bases are in extra-helical conformation bound to the enzyme active site (termed: **TT**^**prot**^ or **CPD**^**prot**^). It should be emphasized that the pdb:1TEZ crystal structure contains a central TT dinucleotide in the enzyme binding pocket although it has been crystallized in the presence of the CPD damage which was photochemically split during the synchrotone exposure^5^. However, we assume that the CPD substrate bound state is very similar since structure determination was performed at very low temperature (<100 K) trapping the structure in the crystallized CPD bound state. This assumption is further supported by the close similartiy of the structure and placement of the intact CPD damage in complex with a homologous repair enzyme (pdb2xrz^6^,) illustrated in Supporting Information, Figure S1. Hence, in order to generate a model of the *E. coli* DNA photolyase in complex with the intact CPD lesion we replaced the central TT dinucleotide by the CPD lesion followed by energy minimisation to remove any sterical overlap. In addition, the original pdb:1TEZ structure also contained a central O5′-C-O3′ group to stabilize the backbone structure during crystallisation which we replaced in the simulations by a regular phosphate group. The equilibration of the complex structures during explicit solvent MD simulations followed the same protocol as described above for the DNA simulations.

### Umbrella Sampling with Hamiltonian replica exchange

Potential of mean force (PMF) calculations of the looping out process of the CPD damage (or two central T nucleotides) from intra-helical to extra-helical states were performed using a pseudo dihedral angle as reaction coordinate. The pseudo dihedral centers that defined the dihedral angle are the heavy atoms of the nucleotides 16,17,18 and 19 (residues on the opposite strand of the damage, center 1), the heavy atoms of nucleotides 1,2,22,23 (center 2), the backbone heavy atoms of the central thymine or CPD nucleotides (center 3) and the heavy atoms of the central thymine or CPD nucleobases (center 4), respectively. The choice of pseudo centers is illustrated in Supporting Information, Figure S2. There are several possible choices to define a pseudo dihedral angle coordinate to induce a flipping out of a damaged base from the intra-helical to the extra-helical state (or vice versa). An advantage of our choice is the definition of each of the four pseudo centers (to define a dihedral) as centers of mass of several atoms. This in turn means that the forces required to flipp a base during the simulations are (equally) distributed on all atoms that define each center which avoids local distortions that can occur if large forces act for example on individual atoms. Indeed, in test simulations we found other pseudo centers to be less suitable (e.g. pseudo centers formed by few atoms nucleotides directly adjacent to the damage) because they resulted frequently in undesired distortions of the DNA (e.g. transient flipping of other bases than the damaged base). To investigate the influence of any global deformation of the DNA (in the absence of the enzyme) simulations were performed on isolated DNA molecules without additional restraints and in another set on DNA positionally restraint to stay close to B-DNA or close to the bound DNA conformation found in the complex with the photolyase repair protein. This was accomplished by weak positional restraints on the backbone of the bases 1 to 4 and 9 to 24 for the undamaged and 1 to 4 and 8 to 23 in the damaged DNA case, respectively. In addition, damage flipping simulations were also performed for DNA in complex with the photolyase protein. The umbrella windows included an angle of −320° to 38° in steps of 2° resulting in a complete 360° coverage for the flipping process. Initialization was run for 100 ps with a torsion force constant of 4000 kcal/(mol rad^2^). Data gathering was performed for 10 ns with a torsion force constant of 400 kcal/(mol rad^2^) and 5000 replica exchange attempts between neighboring umbrella windows every 2 ps (H-REUS-technique, acceptance rate: 0.4). For the simulations including the photolyase the simulation was extended to 20 ns. The intra-helical equilibrium state exhibited an angle of 29° for the **TT/CPD**_**BDNA**_ conformation and 69° for the **TT/CPD**_**1TEZ**_ conformation whereas the extra-helical configuration in the presence of the enzyme (crystal structure **TT**^**prot**^) indicated an angle of −135°. Thus, the required flipping angle is 196° (respectively 156°) in the positive direction as −135° ≡ 225°. The positive direction represents the flipping process through the mayor groove. The flipping through the minor groove requires a dihedral turn of −156° (respectively 204°). The convergence of the calculated free energy change along the reaction coordinate was evaluated in segments of 2 ns per US window (see Supporting Information, Figure S3).

In some of the production US simulations center-of-mass distance restraints (2.5 Å–6.5 Å) to keep the thymine bases relatively close together to improve sampling and to avoid a poor definition of the pseudo torsion dihedral reaction coordinate. The contribution to the free energy change was estimated using free energy perturbation (FEP) (see Supporting Information, Figure S4).

### Calculation of Reaction Rates and Mean First Passage Times

To estimate the rates of the the repair reaction, the diffusion constant *D*(*ξ*) along the one-dimensional reaction coordinate *ξ* was calculated. The diffusion processes along the reaction coordinate can be described by the one-dimensional Smoluchowski equation^44^.


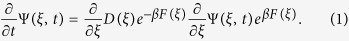


Here ΨI(*ξ, t*) describes the probability density along the one-dimensional coordinate *ξ* at time *t*. The free energy profile *F*(*ξ*) is calculated by US simulations. The following equation relates the local diffusion constant to the time evolution of a specific Umbrella window and is accurate as long as the Umbrella windows are small enough^45^,^46^


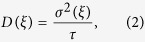


The auto-correlation time was calculated using the method of Hess^47^ which uses block averaging and a double exponential decay fit of the correlation function. The diffusion perpendicular to the reaction coordinate *ξ* influences the local kinetics and thus the local diffusion coefficient. The mean first passage time (MFPT) is the inverse of the specific reaction rate. It can be calculated as a double integral along the one-dimensional reaction coordinate^48^,^49^,^50^,^51^. The intra-helical boundary was set to be reflecting and the extra-helical boundary to be absorbing. This is a reasonable assumption since states in the intra-helical state are quite stable and states in the extra-helical state undergo the subsequent direct repair process and are therefore taken out of the system quickly. The mean first passage time MFPT from the intra- to the extra-helical state is given as


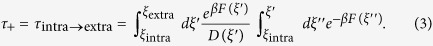


Similarly, the mean first passage time of the inverse process can be calculated as:


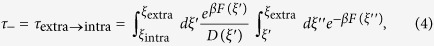


where the probability is integrated along the backwards direction. An example diffusivity profile is indicated in Supporting Information Figure S5. The rates are related as


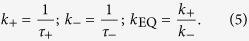


For the flipping processes, the equilibrium rate *k*_EQ_ is related to the free energy difference of the two states by


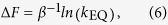


and can be compared to the Umbrella sampling calculations.

## Results and Discussion

### CPD damage transition from intra-helical to extra-helical state in the absence of repair enzyme

**Figure 1:**
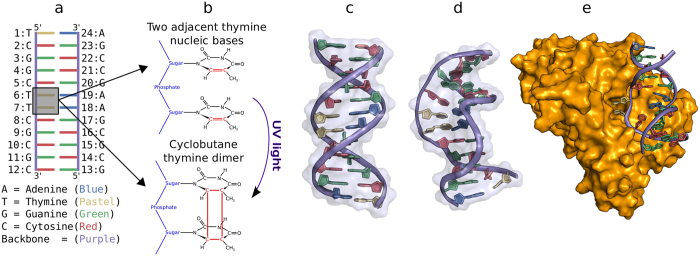
(**a**) Base pair steps and sequence of the DNA oligonucleotides. The duplex molecules included either two standard thymine (T) or a central CPD damage (indicated by an enclosing box in a) opposite to adenine bases. The chemical structure of the central TT or CPD step is indicated in (**b**). MD simulations were either started from standard B-DNA form (**c**), or from an initially deformed DNA structure which was obtained by restraining all backbone atoms (except the damaged nucleotides) to the CPD-damaged DNA crystal structure in complex with the *E. coli* photolyase (pdb-entry:pdb:1TEZ^5^) (**d**). Additional simulations were started from the crystal structure of the damaged DNA in complex with photolyase (orange surface representation in (**e**)).

**Figure 2:**
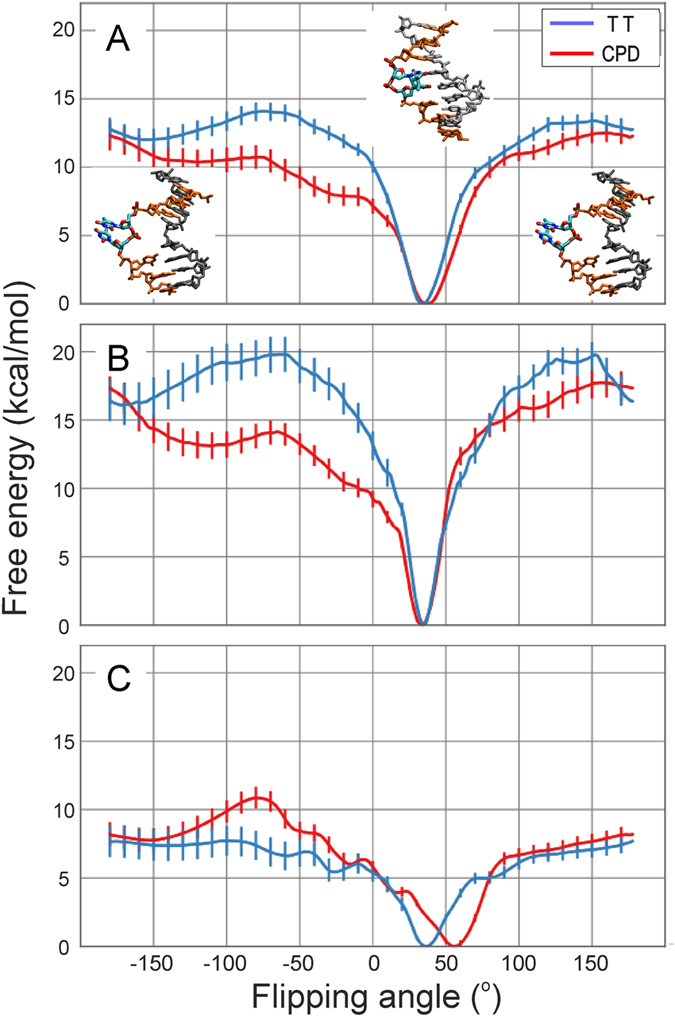
Calculated free energy profiles for TT or CPD damage flipping performed in the absence of the repair enzyme. (**A**) Free energy of the flipping process for simulations on DNA duplexes without additional restraints on DNA. The regions with the central CPD damage in intra-helical conformation (near 30° along the reaction coordinate) and extra-helical (looped out) conformations are illustrated (stick representation, strands in different colors, central damage atom-color coded). (**B**) Calculated free energy for the flipping process with the terminal segments of the DNA restrained to B-DNA form. (**C**) Free energy for flipping process including restraints on terminal segments of the DNA to the repair enzyme bound form (but in the absence of the repair enzyme).

Looping out or flipping of the CPD lesion from an intra-helical to an extra-helical state is necessary to access the active site of a repair enzyme. In order to elucidate the molecular mechanism of this process we performed umbrella sampling (US) free energy simulations along a dihedral reaction coordinate to describe the flipping process (see Methods section for details). For improving the convergence of the simulations frequent Hamiltonian replica exchanges between different umbrella sampling intervals were included (H-REUS technique). A first set of H-REUS simulations was performed on a dsDNA oligonucleotide with a central CPD thymine dimer lesion located at the center of the double helix (Fig. 1). For comparison free energy simulations were also performed on a control dsDNA with a flipping of two neighboring thymines and otherwise identical sequence. During this set of simulations no other restraints besides of the umbrella potential along the dihedral reaction coordinate were applied. The calculated potential-of-mean force for the flipping process indicate an overall lower free energy penalty for flipping the CPD damage compared to a regular central TT sequence (Fig. 2). If one considers the larger range of looped out states relative to inter-helical states the free energy difference between intra-helical vs. extra-helical states amounts to 9 kcal/mol for the TT case vs. 7.5 kcal/mol for the CPD case. This compares qualitatively well with the experimental estimate of 9.5 kcal/mol^13^ for the flipping process of a CPD damaged DNA (an experimental estimate for a TT-flipping is not available). The lower free energy penalty of flipping the CPD lesion is likely due to the fewer hydrogen bonds formed between the damaged bases and the opposite adenine residues and non-optimal stacking in the intra-helical state of the CPD lesion compared to regular DNA with central T:A base pairs. During the flipping process the two thymine bases were restraint to keep an approximately stacked conformation. The free energy of relaxing this restraint was calculated separately for each US window using a free energy perturbation approach (see Supporting Information). On average it amounts to less than 1 kcal/mol for the different simulation intervals (Supporting Information Figure S4b).

It is important to note that the global equilibrium structure of the CPD containing dsDNA differs from the regular TT containing dsDNA. In a previous study we could show that the CPD containing DNA adopts structures during MD simulations that are overall closer to the structure in complex with the repair enzyme (already in the absence of the enzyme) compared to regular dsDNA^22^. In order to elucidate the influence of the global DNA structure on the energetics of the flipping process we performed a second series of H-REUS simulations including restraints to keep segments of the dsDNA oligonucleotides either close to a regular B-form or to the conformation found in the complex with the DNA photolyase (bound DNA structure). It was achieved by weak positional restraints on nucleic acid backbone atoms (atoms P, C5′, C4′, C3′, O5′ and O3′) with respect to either B-DNA or the bound structure excluding the central lesion (or TT sequence) and the flanking nucleotides (see Methods section).

In case of H-REUS simulations including restraints to keep the DNA close to regular B-form the calculated PMFs were qualitatively similar to the PMFs obtained without such restraints. However, the penalty for the flipping process increased by about 2 kcal/mol–3 kcal/mol (Panel 2 in Fig. 2). In contrast, for the simulations including restraints to deform the dsDNA towards the global bound form observed in the DNA photolyase crystal structure the free energy penalty for the flipping process decreased significantly for both the CPD and TT containing dsDNA molecules (Panel 3 in Fig. 2). Interestingly, the decrease is more significant in case of the TT vs. CPD cases presumably because the CPD containing structure adopts a conformation closer to the enzyme bound form already in the absence of the enzyme. In conclusion, the calculations indicate that a global deformation of the DNA towards a the enzyme bound form alone (without accounting for any contacts to the protein) reduces the penalty for the flipping process significantly for both the damaged and the undamaged DNA. A restraining to regular B-DNA causes the opposite effect. Inspection of Fig. 2 reveals another interesting effect. Whereas in unrestrained dsDNA (or restraint to B-DNA) the free energy minimum of the intra-helical state for the CPD lesion was almost identical to the position of the TT motif along the reaction coordinate (Fig. 2) the minimum of the CPD lesion is shifted in the H-REUS simulations with restraints towards the bound form (Panel 3 of Fig. 2).

**Figure 3:**
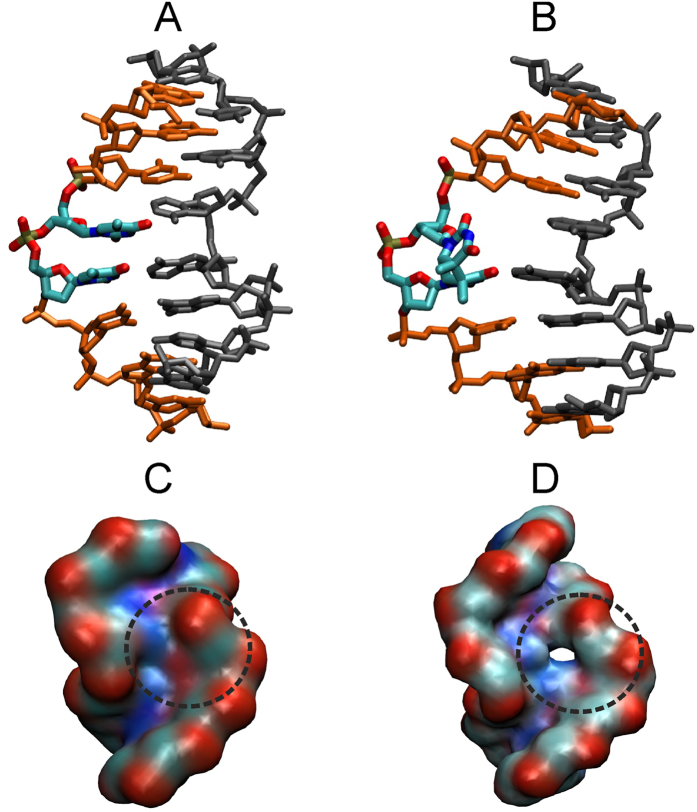
Free energy minima for the central damaged or undamaged DNA obtained during free energy simulations of DNA in near bound conformation. (**A**) The free energy minimum obtained for the regular TT case near 30° along the flipping reaction coordinate indicates a base-paired central arrangement (strands in orange and grey, central TT nucleotides in atom color code). (**B**) The free energy minimum obtained for the CPD damage is located at 50° along the reaction coordinate and indicates a structure that is partially looped out into the major groove, at same time creating sterically accessible space on the minor groove surface of the DNA. (**C**) View into the minor groove of the free energy minimum obtained for regular DNA globally restraint to the repair enzyme bound form (atom-color coded surface representation). (**D**) Same as (**C**) but for the CPD damaged case clearly indicating sterically accessible space at the CPD damage site. The protein binding region (with a central Pro-residue fitting into the sterical accessible space).

The shift of the minimum of the internal configuration from 30° to 60° in the damaged case can be attributed to the rigidity of the damaged dimer not being able to conform to the overall bend induced by the external positional restraint. The regular DNA although under strain still keeps a hydrogen bonded geometry of the central base pairs at a 60° looping out dihedral angle (see Fig. 2 and Fig. 3) whereas the CPD lesion adopts a partially flipped conformation towards the DNA major groove at the same looping dihedral angle (see Fig. 2 and Fig. 3). This also creates a cavity on the minor groove side of the DNA which corresponds to the side that is bound by the protein in the complex with the DNA photolyase Fig. 3.

### Umbrella sampling in presence of Photolyase protein

**Figure 4:**
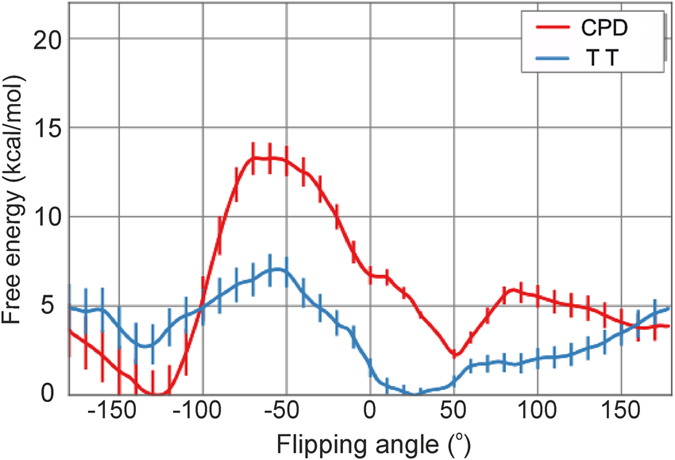
Calculated free energy change for the TT and CPD flipping process in the presence of the repair enzyme.

**Figure 5:**
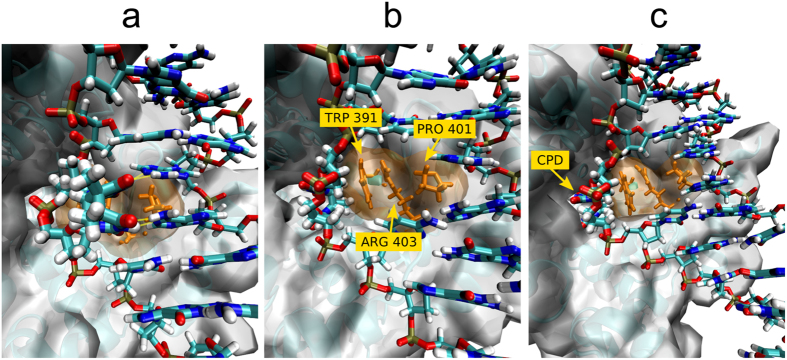
Snapshots observed during the flipping process of the CPD damage in complex with the photolyase enzyme. (**a**) CPD (bold sticks) in the intra-helical conformation with the maximum number of hydrogen bonds formed with the adenine bases on the opposite strand (H-bonds indicated as yellow sticks). The Pro401 (orange sticks) of photolyase at the minor groove of DNA sterically contacts the CPD bases. (**b**) Upon movement of the CPD lesion between DNA minor groove and protein during free energy simulations for flipping towards the minor groove the flipped CPD clashes with residues Trp391, Arg403 and Pro401 (all orange) which increases the barrier for flipping along the minor groove. (**c**) Location of the CPD at the enzyme active site pocket of the photolyase after completion of the flipping process.

In addition to H-REUS simulations of isolated dsDNA oligonucleotides, simulations were also performed with the DNA in complex with the DNA photolyase repair enzyme. Simulations were started from a known crystal structure of the CPD containing DNA-repair enzyme complex (including the FADH cofactor). The presence of the protein significantly modulated the calculated PMF curves along the reaction coordinate (Fig. 4). The calculated PMF curves indicate two free energy minima, one corresponds to the intra-helical state (at 30°) and the second minimum is located in the extra-helical regime and corresponds to the localization of the CPD lesion (or TT motif) in the active site of the enzyme (at −130°). For the CPD lesion this extra-helical conformation is of lower few energy compared to the intra-helical state by −2 kcal/mol and for the TT case the extra-helical state is still less stable by 2 kcal/mol compared to the intra-helical state. Interestingly, the bound enzyme result in a lowering of the free energy barrier for flipping both the CPD lesion or the TT bases towards the major groove but yields still a relatively high barrier for the CPD case for flipping along the minor groove. It is due to contacts of the DNA at the minor groove side to the protein which results in significant sterical barriers for the bulky and rigid CPD lesion to glide between the minor groove and the protein surface from the intra-helical to the extra-helical state. The steric clashes with the protein involve residues TRP391, ARG403, and PRO401 contacting the minor groove of the bound DNA (Fig. 5).

In contrast, the TT motif although restraint to a stacked state is still much more flexible and can better adapt to the space between protein and DNA during the flipping process along the major groove. For the flipping process towards the major groove the free energy penalty is smaller even compared to the flipping with a DNA deformed towards the bound form (by approx. 1 kcal/mol–2 kcal/mol due to additional protein-DNA contacts (pushing from the minor groove and attractive interactions to move towards the active site cavity in the enzyme).

### Reaction Rates and Mean First Passage Times

The umbrella sampling simulations were also used to estimate the mean first passage times and kinetics of the nucleotide flipping processes (see Methods) by calculating the diffusivity along the reaction coordinate. The differences in the rates of the flipping reaction of the damaged and undamaged DNA (especially in the presence of the protein) are more pronounced as the free energy differences since both free energy landscape and local diffusion constants along the reaction coordinate influence the kinetics. The calculated diffusivity shows large fluctuations (one order of magnitude) and it should be emphasized that the calculated mean first passage times are only estimates. The kinetics are not only influenced by the energy landscape but also by the shorter pathway to reach the extra-helical state in case of the CPD damage (the intra-helical equilibrium state is in this case already shifted towards the extra-helical state by around 20°). According to the calculated mean first passage times, the CPD containing DNA in the presence of the repair enzyme flips from the intra-helical to the extra-helical state in roughly 120 μs whereas the TT containing DNA needs on average much longer (about 3900 μs, Table 1). For similar repair enzyme systems such as glycosylases, typical residence times of the enzyme at every nucleotide site of 50 μs have been measured^52^. During this time the repair procedure needs at least to be initiated. This time is in a similar order as the calculated estimate for the CPD damage flipping process but much shorter than the mean first passage time for the flipping process of an undamaged TT motif. In order to check the influence of the calculated diffusivity profile on the mean first passage times it was replaced by one average (constant) diffusion constant along the reaction coordinate. This leads to smaller calculated mean first passage times (because the reduced roughness in the diffusion landscape increases the reaction rate, similar to the roughness of the potential as described by Zwanzig^50^). However, the reduction is smaller than a factor 5 and the order of the calculated mean first passage times does not change (data not shown).

## Conclusions

Extensive free energy simulations have been employed to elucidate the recognition mechanism of CPD damages in DNA. This includes the separate analysis of the contribution of global DNA deformation and of a repair enzyme bound to the damaged site. The simulations indicate that in the unbound state the free energy penalty for flipping a CPD lesion from the intra-helical state to an extra-helical conformation is lower compared to regular undamaged DNA. However, the calculated free energy penalty of 8 kcal/mol is still too high to allow for frequent spontaneous flipping events. The high free energy penalty leads to a low effective concentration of extra-helical CPD lesions relative to intra-helical states of 10^−6^. We demonstrated for the first time that the deformation of the DNA alone towards the global bound structure results already in a significant lowering of the calculated free energy penalty for flipping without involving any direct contacts of the protein with the damaged bases. Molecular modelling studies have suggested an influence of global DNA changes for base pair opening but the role for damage recognition and damage flipping in explicit solvent simulations has so far not been indicated^53^,^54^.

Such global deformations of DNA involving undertwisting, bending and often minor groove opening have been observed in many crystal structures of damaged DNA in complex with repair enzymes indicating that global deformation may also facilitate damaged base opening transitions in other repair processes. This conclusion is further supported by recent experimental studies on the Rad4/XPC nucleotide excision repair complex which suggests a a twist-open mechanism damage recognition^55^. Similarly, in the case of the Rad14 (homologue of the XPA protein that recognizes damaged DNA in eukaryotes to initiate the nucleotide excision repair pathway) it has been found that DNA binding induces a strong DNA deformation which results in increased damage accessibility (partial looping out) without contacts between XPA and the DNA damage^56^.

We found also that the calculated free energy penalty for the CPD flipping to the major groove is further lowered by the presence of the protein and contacts of protein and DNA. However, only for the CPD damage (and not the undamaged TT motif) the free energy of the extra-helical active site bound state is lower than the free energy of the intra-helical state.

Together with the estimates on the kinetics of the flipping processes a model for the recognition and repair process can be derived. As was shown previously, CPD damaged DNA can adopt conformations globally closer to the bound conformation compared to undamaged DNA^22^. It explains the significantly higher affinity observed experimentally for binding of CPD containing DNA to the photolyase repair enzyme^4^,^57^,^58^,^59^. It suggests that the initial encounter binding is partially based on conformational selection but also requires an induced fit to complete the DNA deformation towards a conformation that fits to the repair enzyme. Already this encounter process strongly favors binding of a damaged site and not of regular DNA. Another important finding of the present study is the identification of a putative intermediate encounter structure of the CPD-damage with a partially looped out conformation. This intermediate fits very well to the protein binding surface (in contrast to the binding of regular DNA) and adds to the much better binding of CPD-damaged DNA compared to regular DNA.

In addition, the encounter binding step coupled to a global DNA deformation already significantly lowers the penalty for the flipping process. Subsequently, the formation of specific contacts to the protein further facilitate the flipping of the damage towards the mayor groove. Although the barrier for this process is similar for damaged as well as undamaged DNA the transition is overall only favorable for flipping the CPD lesion. The photo-chemical cleavage of the CPD lesion results in the formation of two thymine bases that now can flip back into an intra-helical state involving small barriers both for flipping through the minor or major grooves of the DNA. This allows an efficient product release after the repair reaction with barrier heights of less than 5 kcal/mol. The model places the main selection step for damaged DNA at the initial phase which corresponds to recognition and the necessary DNA deformation for precise placement of the damaged DNA at the vicinity of the active site. Given the low abundances of CPD damaged sites relative to regular TT motifs a selection at an initial step of the process is more efficient compared to a distinction at a later stage, e.g in the active site of the enzyme. The conclusions are also strongly supported by our estimates for the mean first passage times for flipping which predict a much more rapid flipping of the CPD damage in the presence of the repair enzyme in the same order as the estimated average time a DNA site is bound by the enzyme for undamaged DNA this time was found to be much larger than mean residence time of the repair enzyme at a DNA site. It is likely that similar recognition mechanisms are the basis of other repair processes which also involve encounter binding with the DNA adopting a deformed structure and subsequent facilitated flipping of the damaged bases into the enzyme active site^60^,^61^,^62^,^63^.

## Additional Information

**How to cite this article:** Knips, A. and Zacharias, M. Both DNA global deformation and repair enzyme contacts mediate flipping of thymine dimer damage. *Sci. Rep.* **7**, 41324; doi: 10.1038/srep41324 (2017).

**Publisher's note:** Springer Nature remains neutral with regard to jurisdictional claims in published maps and institutional affiliations.

## Supplementary Material

Supplementary Information

## Figures and Tables

**Table 1:** Calculated Mean First Passage Times for the flipping reaction from the intra- to the extra-helical state and *vice versa*.

Simulation	*τ*_+_[s]	*τ*_−_[s]
**CPD**_**BDNA**_	3.8 × 10^1^	1.7 × 10^−6^
**TT**_**BDNA**_	2.0 × 10^1^	4.6 × 10^−7^
**CPD**_**1TEZ**_	4.5 × 10^−3^	1.9 × 10^−7^
**TT**_**1TEZ**_	1.6 × 10^−1^	5.2 × 10^−7^
**CPD**^**prot**^	1.2 × 10^−4^	1.7 × 10^−2^
**TT**^**prot**^	3.9×10^−3^	3.7×10^−3^
