# Supplementary material for: Both DNA global deformation and repair enzyme contacts mediate flipping of thymine dimer damage
Source: Sci Rep. 2017 Jan 27;7:41324. doi: 10.1038/srep41324 (PMC5269681; doi:10.1038/srep41324)
Supplement: Supplementary Information [file srep41324-s1.pdf]

SUPPORTING INFORMATION: Both DNA global deformation and repair enzyme contacts mediate flipping of thymine dimer damage

Alexander Knips[a] and Martin Zacharias*,[a]

[a]*Physik-Department T38, Technische Universität München, James-Franck-Str. 1, D-85748 Garching Germany*

Definition of a pseudo-dihedral angle for damage flipping

The pseudo-dihedral angle used as reaction coordinate was defined by four centers of mass illustrated in Figure S2. Each of the centers consisted of the heavy atoms of several nucleotides to optimally distribute the restraining forces on many atoms. Test simulations indicated that this minimizes the local perturbation of the structure during the umbrella sampling simulations.

Convergence of Umbrella Sampling Simulations

Contribution of restraining two neighboring thymines for the simulation of undamaged DNA

The free energy contribution of the distance restraint between the two thymine bases during umbrella sampling was calculated using free energy perturbation. It was accomplished by running simulations in the absence of the restraint and treating the application of the restraining potential as a perturbation. To improve the accuracy the Bennett acceptance ratio (BAR) method

*Corresponding author. Tel.: +49 89 289 12335, Fax.: +49 89 289 12444
E-mail: martin.zacharias@ph.tum.de

[1] was employed. For most umbrella simulation windows the calculated contribution was less than 1 kcal/mol (average: 0.47 kcal/mol, Figure S4a). As a further control the free energy along the reaction coordinate was calculated for the protein-bound case using different types of restraints and one series of simulations without restraints on the thymine bases (Figure S4b). The calculated curves deviated from a mean free energy curve by less than 1 kcal/mol.

Diffusion constant along the reaction coordinate

In addition to the free energy change, the diffusivity profiles along the one-dimensional reaction coordinate were calculated (shown for the case of flipping the CPD damage in the presence of the repair enzyme, Figure S5).

To check the H-REUS sampling simulation for convergence, we split the output files of the measurements of the specific reaction coordinate into 5 time intervals and calculated the PMF for each of those time intervals separately. By plotting the resulting free energy curves trends and convergence can be checked for the US simulations (see Figure S3).

References

[1] C. Steffen, K. Thomas, U. Huniar, A. Hellweg, O. Rubner, and A. Schroer, "Unorthodox Uses of Bennett's Acceptance Ratio Method," *J. Comput. Chem.*, vol. 31, no. 16, pp. 2967–2970, 2010.

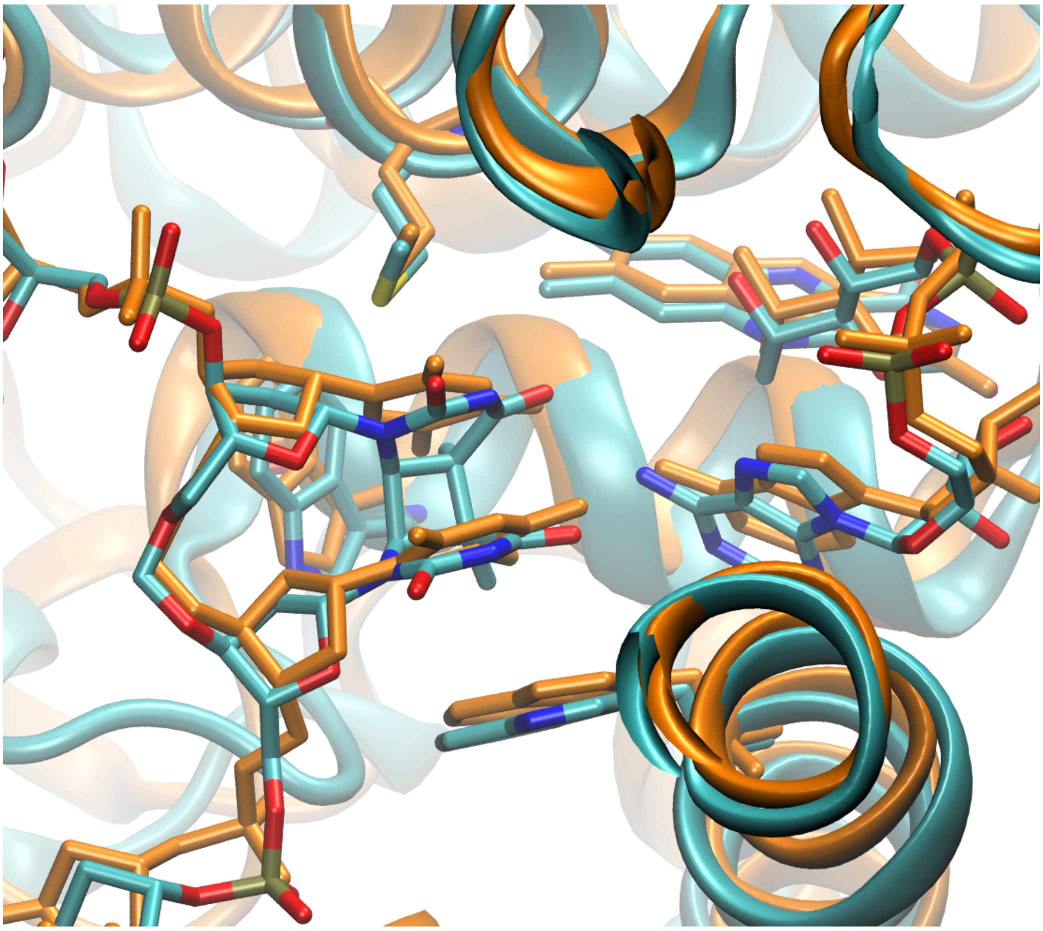

Figure S1: Close view into the damage lession binding site of the E.coli Photolyase in complex with a central TT dinucleotide in the substrate binding pocket (pdb1TEZ, orange Cartoon and stick representation of selected residues in the active site). The structure of the homologous enzyme from Methanosarcina Mazei in complex with an intact CPD damage(pdb2RXZ) superimposed with respect to the protein backbone is indicated for comparison (atom-color coded sticks and cyan Cartoon). The root-mean-square deviation between atoms of the CPD damage and the TT dinucleotide heavy atoms is smaller than 1 Å.

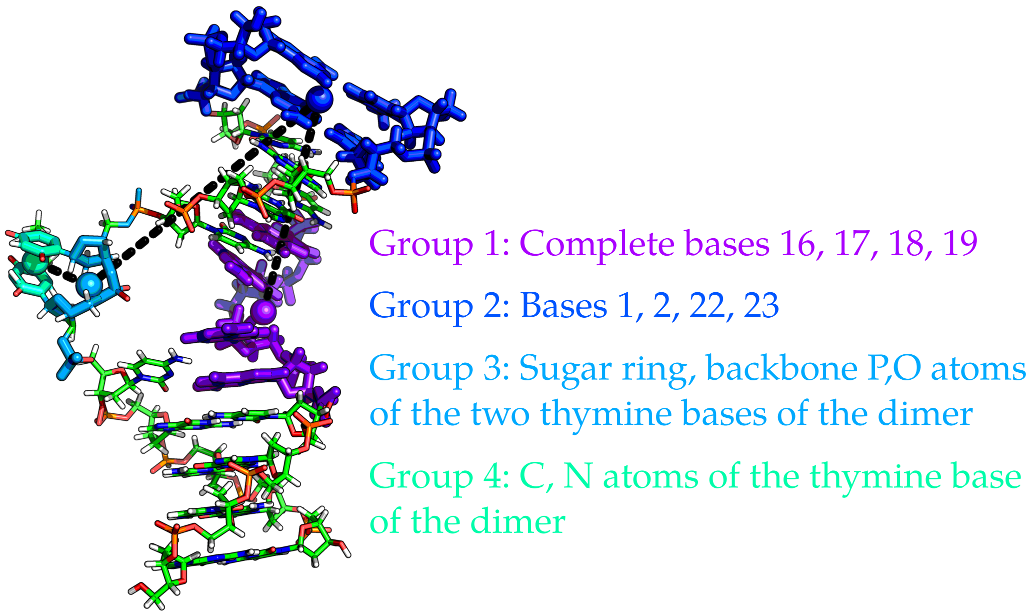

Figure S2: The reaction coordinate for the flipping process is defined as a dihedral angle formed by four centers-of-mass of groups of atoms (colored-coded). The first group includes the heavy atoms of the nucleotides 16,17,18 and 19 (residues on the opposite strand of the damage), the heavy atoms of nucleotides 1,2,22,23 form center 2, the backbone heavy atoms of the central thymine or CPD nucleotides define center 3 and the heavy atoms of the central thymine or CPD nucleobases form center 4, respectively.

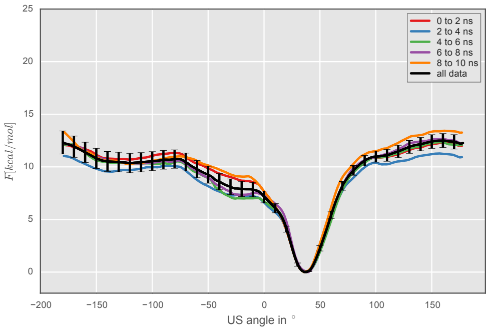

(a) $\mathbf{CPD_{BDNA}}$

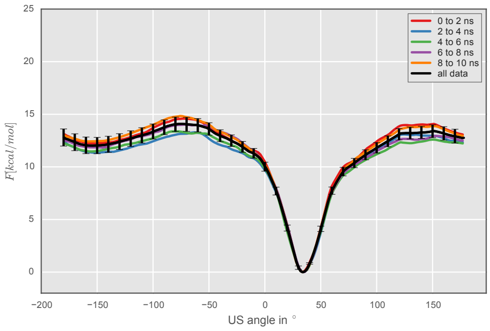

(b) $\mathbf{TT_{BDNA}}$

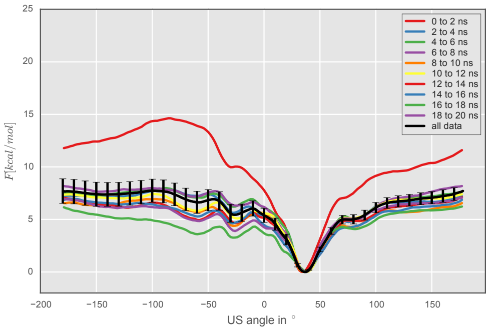

(c) $\mathbf{CPD_{1TEZ}}$

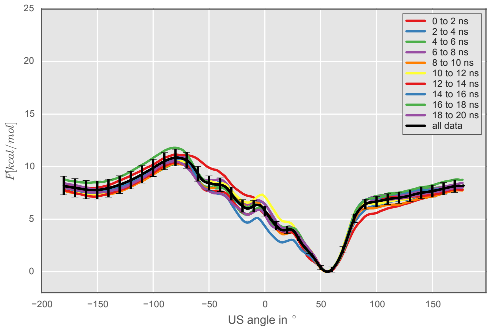

(d) $\mathbf{TT_{1TEZ}}$

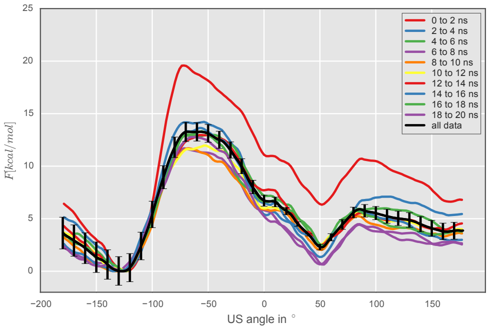

(e) $\mathbf{CPD^{prot}}$

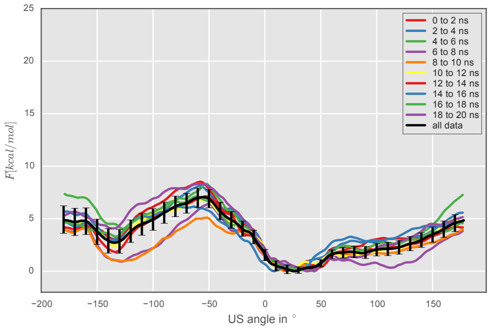

(f) $\mathbf{TT^{prot}}$

Figure S3: Convergence free energy simulations illustrated as free energy plots obtained from different time segments from each US interval for the unrestraint DNA cases (A). For the simulations with restraints on the global DNA structure (B) and in the presence of repair protein (C) simulations have been extended to 20 ns.

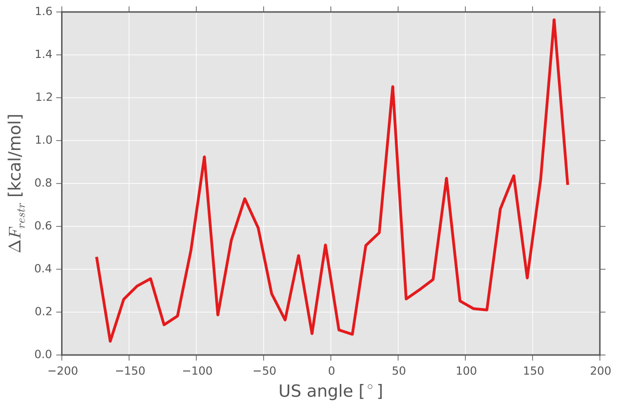

(a)

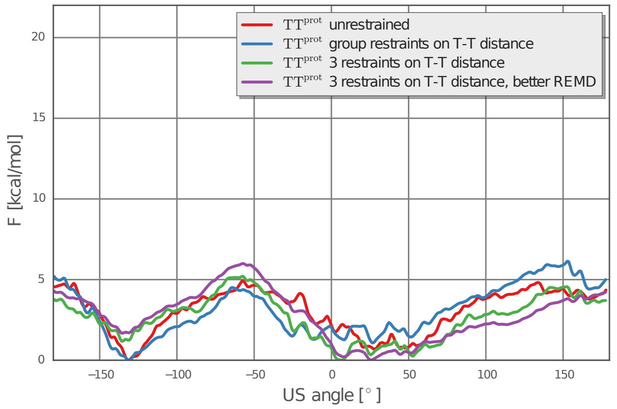

(b)

Figure S4: (a) Free energy contribution of restraining the configuration of the central thymine residues in a stacked conformation during US simuations. The free energy contribution was estimated from running simulations without restraints between centers-of-mass of the two thymine bases and treating the addition of the restraining potential as a perturbation (FEP approach). (b) As a second control the influence of the distance restraint on the free energy along the reaction coordinate was directly calculated using different types of restraints and one series of simualtions without restraints on the thymine bases.

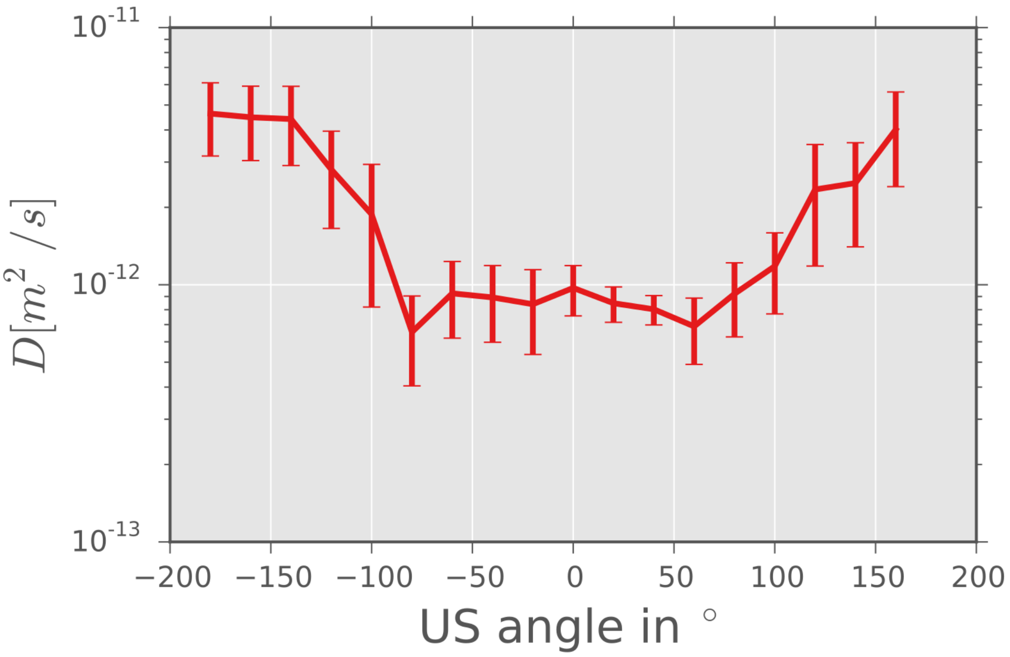

Figure S5: Calculated diffusion constants obtained for the US simulation windows (CPD damage in complex with repair enzyme. Mean diffusion constants and standard deviations were calculated as window averages over 6 consecutive US intervals.
